# What smells? Developing in‐field methods to characterize the chemical composition of wild mammalian scent cues

**DOI:** 10.1002/ece3.6224

**Published:** 2020-04-12

**Authors:** Cynthia L. Thompson, Kimberly N. Bottenberg, Andrew W. Lantz, Maria A. B. de Oliveira, Leonardo C. O. Melo, Christopher J. Vinyard

**Affiliations:** ^1^ Department of Biomedical Sciences Grand Valley State University Allendale MI USA; ^2^ Department of Chemistry Grand Valley State University Allendale MI USA; ^3^ Departamento de Morfologia e Fisiologia Animal Universidade Federal Rural de Pernambuco Recife Brazil; ^4^ Department of Anatomy & Neurobiology Northeast Ohio Medical University Rootstown OH USA

**Keywords:** chemical ecology, fruit odor, marmoset, olfactory cues, portable GC‐MS, scent marking

## Abstract

Olfactory cues play an important role in mammalian biology, but have been challenging to assess in the field. Current methods pose problematic issues with sample storage and transportation, limiting our ability to connect chemical variation in scents with relevant ecological and behavioral contexts. Real‐time, in‐field analysis *via* portable gas chromatography–mass spectrometry (GC‐MS) has the potential to overcome these issues, but with trade‐offs of reduced sensitivity and compound mass range. We field‐tested the ability of portable GC‐MS to support two representative applications of chemical ecology research with a wild arboreal primate, common marmoset monkeys (*Callithrix jacchus*). We developed methods to (a) evaluate the chemical composition of marmoset scent marks deposited at feeding sites and (b) characterize the scent profiles of exudates eaten by marmosets. We successfully collected marmoset scent marks across several canopy heights, with the portable GC‐MS detecting known components of marmoset glandular secretions and differentiating these from in‐field controls. Likewise, variation in the chemical profile of scent marks demonstrated a significant correlation with marmoset feeding behavior, indicating these scents’ biological relevance. The portable GC‐MS also delineated species‐specific olfactory signatures of exudates fed on by marmosets. Despite the trade‐offs, portable GC‐MS represents a viable option for characterizing olfactory compounds used by wild mammals, yielding biologically relevant data. While the decision to adopt portable GC‐MS will likely depend on site‐ and project‐specific needs, our ability to conduct two example applications under relatively challenging field conditions bodes well for the versatility of in‐field GC‐MS.

## Graphical abstract

Olfactory cues play an important role in mammalian biology, but have been challenging to assess in the field. We field‐tested the ability of portable GC‐MS to support two representative applications of chemical ecology research. Despite trade‐offs, portable GC‐MS represents a viable option for characterizing olfactory compounds used by wild mammals, yielding biologically relevant data.

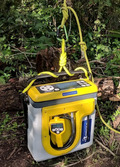

## 1 INTRODUCTION

Scents are essential in the sensory repertoire of mammals. Olfactory cues are used by wild animals to gain information across a wide range of ecological and social contexts, such as dominance interactions, competition, sexual interactions, foraging, and predator/prey detection (e.g., Boulet, Charpentier, & Drea, 2009; Laska et al., 2005; Vaglio, Minicozzi, Bonometti, Mello, & Chiarelli, 2009). Variation in the chemical composition of olfactory cues can carry information to recipients, such as individual information about the signaler or suitability of food items (Apps, 2013; Crawford & Drea, 2015; Smith, 2006). Despite the recognized significance of scents, research on olfaction in wild mammals lags behind other senses (Nevo & Heymann, 2015; Semple & Higham, 2013). This is partially due to a dearth of feasible methods to measure how scents vary in real time, under the natural ecological‐ and evolutionary‐relevant conditions experienced by wild mammals.

Gas chromatography–mass spectrometry (GC‐MS) has long been the standard method for characterizing the chemical composition of olfactory compounds (Drea et al., 2013; Soso, Koziel, Johnson, Lee, & Fairbanks, 2014). However, this technology has been limited to laboratory settings due to its weight, bulk, and need for a stable power source, making it incompatible with the minimalistic, rugged nature of many field sites (Drea et al., 2013). Studies gathering olfactory compounds under wild contexts have often been required to store and transport samples for GC‐MS analysis (Drea et al., 2013; Nair, Shanmugam, Karpe, Ramakrishnan, & Olsson, 2018; Valenta et al., 2015). Unfortunately, this transportation creates problems due to degradation and/or evaporation of the volatile organic compounds known to be a part of olfactory cues (Drea et al., 2013; Nair et al., 2018). While sorbent tubes have been touted as a recent innovation that can overcome these issues (Kücklich et al., 2017; Weiß et al., 2018), these tubes do not provide the long‐term stability needed for many field studies (Kallenbach et al., 2014; Nair et al., 2018). For instance, Koziel et al. (2005) found that recovery of volatile organic compounds stored in sorbent tubes was reduced by 88.3% after only 120 hr at room temperature. Others have reported acceptable preservation for up to two weeks, but only when stored at 5–10°C (Harshman et al., 2016; Kang & Thomas, 2016; Van der Schee et al., 2012). Likewise, a number of studies have revealed retention biases for different compound classes following storage (Harshman et al., 2016; Kallenbach et al., 2014; Kücklich et al., 2017; Nair et al., 2018). Hence, while these tubes may suffice for short‐term storage at ambient temperature, they are not a viable option for a large portion of field studies, particularly those that involve significant travel or lack the infrastructure to refrigerate or freeze samples.

Beyond preservation issues, research on olfaction in wild mammals has often lacked a real‐time, ecological and behavioral context for the scents collected. Previous studies have captured animals to collect secretions by expressing glands (e.g., Drea et al., 2013; Spence‐Aizenberg, Kimball, Williams, & Fernandez‐Duque, 2018; Stoffel et al., 2015; Zidat et al., 2018). This approach limits the scientific questions that can be addressed, as there is no relevant relationship between the scent gathered and the behavior of signalers or recipients utilizing that scent. These types of discrete odor collection events pose difficulties for testing longitudinal questions about olfaction (other than on gross scales *via* recapture: see Hayes, Morelli, & Wright, 2006). The inability to measure scents in real time, including how they vary with the dynamic and multifaceted contexts in which animals use scent, has hindered our ability to connect olfactory cues with the informational content animals are utilizing (Apps, 2013; Semple & Higham, 2013).

**FIGURE 1:**
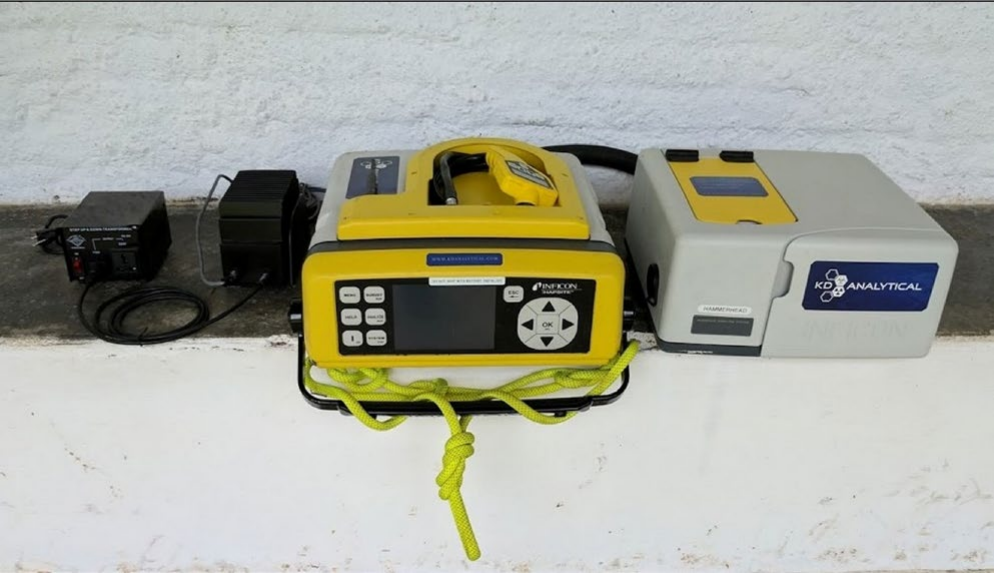
Inficon Hapsite Portable GC‐MS (center) with headspace sampling systems (right), which enables analysis of solids and liquids. At left: AC power unit and voltage converter. The airprobe is shown in storage position on top of the Hapsite. Details of the fastened hoisting rope are outlined in Appendix S1

Portable GC‐MS (Diken et al., 2012; Hall & Mulligan, 2014) holds potential to advance olfactory research on free‐ranging animals. Portable GC‐MS units are field‐durable and provide results comparable to benchtop laboratory systems, as validated by studies testing samples on both laboratory and portable models (Beckley, Gorder, Dettenmaier, Rivera‐Duarte, & McHugh, 2014; Cal EPA, 2004; Einfeld, 1998; Inficon, 2011). The model we focus on here, the Hapsite Smart Plus ER (Inficon) (Figure 1), weighs 19 kg (dimensions: 46 × 43 × 18 cm) and can operate on battery power, enabling significant portability in the field. However, this portability necessitates some modifications: run time is shorter, the maximum temperature obtained is lower than for benchtop GC‐MS, and the mass spectrometer has a poorer detection limit. These devices are consequently less effective at collecting higher mass, nonvolatile compounds and are less sensitive toward compounds present in low quantities. Nonetheless, previous validation studies using mammalian glandular secretions from captivity found that 94% of compounds detected with the Hapsite were identical to those found with benchtop analysis, demonstrating efficacy of this device (Kücklich et al., 2017). However, 100% of these compounds were classified as volatile, with no nonvolatile compounds detected. As such, field testing is needed to assess the utility of these devices to measure compounds biologically relevant to mammalian chemical ecology under field conditions (Kücklich et al., 2017). Field testing is also necessary to assess the practical ability of this method to overcome logistical challenges unique to the field, such as accessing samples and controlling for compounds present in the background environment.

Common marmoset monkeys (*Callithrix jacchus*), an arboreal Neotropical primate, provide an advantageous model for field testing this equipment since much is known about their use of olfaction from both the field and laboratory (e.g., Lazaro‐Perea, Snowdon, & Fátima Arruda, 1999; Oliveira & Macedo, 2010; Smith, 2006; Ziegler, Peterson, Sosa, & Barnard, 2011), and their scent secretions have previously been characterized via GC‐MS (Kücklich et al., 2017; Smith, Tomlinson, Mlotkiewicz, & Abbott, 2001). Wild marmosets deposit scent marks in numerous social and ecological contexts, including when gouging trees and lianas to feed on exudates (Lazaro‐Perea et al., 1999). The spatially stationary and renewable nature of exudates allows for revisitation of food sites and the use of long‐term signals, such as scent marks, which may provide persistent information about food sources that could aid foraging decisions (Thompson, Blanck, Pearson, Scheidel, & Vinyard, 2018), as has been demonstrated for other mammalian food sources such as ripe fruits (Rodríguez, Alquézar, & Peña, 2013; Valenta et al., 2015). Lastly, marmosets are relatively easy to follow and observe, allowing integration of information on olfactory cues with ecological and behavioral context. Linking variation in the chemical composition of scents with signalers’ and recipients’ physiology and behavior can help overcome the current hurdles preventing real‐time, longitudinal studies of olfactory communication of wild mammals.

We field‐tested whether portable GC‐MS can produce meaningful data on the olfactory cues utilized by free‐ranging mammals, within the context of foraging by common marmosets. We trialed two applications of portable GC‐MS relevant to both marmoset, and more broadly, mammalian chemical ecology: (a) collecting information on the chemical composition of marmoset scent marks, and relating this composition to social and ecological context, and (b) characterizing differences in the olfactory signature of foods eaten by marmosets, a cue which could be used in food selection. Both of these topics have garnered considerable research interest (Drea et al., 2013; Kean, Müller, & Chadwick, 2011; Nevo, Heymann, Schulz, & Ayasse, 2016; Rodríguez et al., 2013), making them representative applications to test portable GC‐MS technology. Additionally, we will outline practical methodological considerations for using these units under field conditions, to provide guidance for researchers interested in adopting this technology.

## 2 MATERIALS AND METHODS

### 2.1 Study site

Research was conducted at Tapacurá Ecological Field Station, Pernambuco, Brazil (08°03S, 35°12W), within the Atlantic Coastal forest (Moura, 2019; Moura, Júnior, & El‐Deir, 2012). Permission to conduct research was provided by the Brazilian Science and Technology Minister (Portaria MCTIC N°7.423/2017), Brazilian Minister of the Environment (License SISBIO N°58967‐2 ICMBio/MMA), and Ethical Committee (License N°49/2017 CEUA/UFRPE).

### 2.2 Application 1: Scent mark sampling

#### 2.2.1 Accessing scent marks

We initially trialed different field setups for monitoring animals, transporting the portable GC‐MS, and accessing scent marks to determine what approach would functionally work best in the field. We ultimately adopted a “home base” approach, in which the device was housed at a location where it could be readily plugged in to preserve battery life and retrieved for sampling. For our field site, this home base was near an area that marmosets frequently fed on exudates. We continuously monitored this home base and the surrounding ~100 m radius for marmoset activity, and followed all observed animals until they left this area. As our goal was to determine feasibility, this approach facilitated collecting as many samples as possible (by focusing on a feeding area where scent marks were more likely to occur), while preserving battery life of the device (see discussion).

When scent marking was observed, the location of the mark was noted, and researchers waited for animals to leave the area prior to accessing scents. This limited the impact of sampling on marmoset behavior, although greater waiting periods could lead to sample degradation, and hence, fewer compounds being detected. To test for such an effect, we measured the latency to sample collection (i.e., minutes between sample deposition and sample collection, $\bar{x}$ = 22 min) and conducted a bivariate linear regression on sample richness (the number of compounds detected in scent marks). We did not find a significant effect of waiting period on sample richness (*β* = −0.01, *p* = 0.930, *R* ^2^ < 0.001). We collected *N* = 64 scent mark samples, all of which were anogenital marks that occurred in the context of exudate feeding. Scent marking of gouge holes was defined as animals placing their anogenital region level with the exudate hole, pressing the pelvic region close to the tree, and engaging in repetitive rubbing movements.

The logistics of accessing scent marks varied depending on height in the canopy and forest structure. Our setup to access marks fell into three categories: (a) marks within standing height that did not require additional processing to access, (b) marks accessed via ladder (~1–3 m off ground), and (c) marks higher in the canopy that were accessed with both ladders for researchers to access the sample and a rope system to lift the GC‐MS into the tree (further details in Appendix S1). We were successful in collecting scent marks from a range of heights within the forest canopy (3.1% of samples < 1 m; 78.1% within 1–5 m; 18.8% within 5–10 m). For all approaches, the device was returned to the home base for the remainder of sample analysis to conserve battery power. Our home base approach also allowed us to conduct all‐occurrence behavioral sampling at gouged exudate holes within the (~100 m radius) monitored feeding area, with multiple observers present during all day‐light hours. Details on the sex and individual identity of scent marking animals were not able to be consistently collected. However, after sampling, a small marking was placed at exudate holes to facilitate identification, and we recorded all feeding visits and feeding‐related scent remarks by animals at these holes. We also measured hole dimensions to calculate gouge hole volume, a proxy for use intensity since repeated feeding and gouging leads to larger holes (Thompson et al., 2014).

#### 2.2.2 Sampling scent compounds

We sampled airborne compounds emanating from scent marks (following Perrin, Rasmussen, Gunawardena, & Rasmussen, 1996; Rasmussen & Wittemyer, 2002). Collecting liquid samples proved infeasible due to the low volume of scent marks produced by marmosets, mirroring previous captive studies (Smith, Abbott, Tomlinson, & Mlotkiewicz, 1997). Airborne compounds represent the cue that recipient animals smell when approaching scent marks (with nonvolatiles being accessed through muzzle rubbing, licking or similar behaviors) and have been shown to provide a biologically relevant variable for measuring olfactory signaling (Rasmussen & Wittemyer, 2002; Weiß et al., 2018).

To concentrate scent compounds prior to sampling, we inverted a sealed stainless steel funnel over scent marks for 5 min (Perrin et al., 1996; Rasmussen & Wittemyer, 2002). All funnels were double washed with acetone and dried in an oven prior to use. We tested three funnel sizes (7 × 4 mm, 5 × 3 mm, and 4 × 2 mm) to determine which was most effective. No consistent differences were found in sample richness based on funnel size (Kruskal–Wallis test: *χ* ^2^ = 4.62, *p* = 0.100), and so all data were pooled. After concentrating samples, the opening of the funnel's stem was exposed and the Hapsite's airprobe (Figure 1) was used to collect the sample. Air was sampled for 2 min, and analysis began immediately afterward and lasted 25 min.

Samples were analyzed with the Hapsite Smart Plus (Inficon). The device possesses a carbon concentrator and a 100% methylpolysiloxane stationary phase GC column (30 m × 0.32 mm ID × 1.0 μm film). Carrier gas was ultra‐high purity nitrogen with a 15 L/s nonevaporable getter pump and 0.2 L/s sputter‐ion pump. Electron ionization mode was used at 70 eV. The MS scan range was 41–300 *m*/*z*. The temperature ramp of the GC oven was as follows: 50°C for 7 min, climbing to 110°C across 10 min, then up to 180°C across 4 min 40 s, and holding at 180°C for an additional 3 min 20 s.

Once GC‐MS analysis of a scent mark was complete, we then immediately collected a matched control for each sampled scent mark. Controls were taken on the same tree or liana within 1 m of the original sample, on the opposite side of the branch/trunk from the scent mark. Selected control areas were visually free of fungus, scars, spikes, or other obvious irregularities. The procedures described above (funnels, heat ramps, etc.) were carried out identically for controls and samples.

### 2.3 Application 2: Food odor sampling

Food samples were collected from exudate species that marmosets were observed feeding on during the study period (Table 1). Samples were collected directly from plants in 40 ml Supelco GC‐MS vials (Cat#27180). All tubes and collection equipment were washed following the above procedure for funnels.

**Table 1:** Species, sample size, and chemical richness (number of distinct compounds) of sampled exudates eaten by marmosets

Species	Family	*N*	$\bar{x}$ chemical richness^a^	Range chemical richness^a^
*Acacia paniculata*	Fabaceae	5	14.2	9–20
*Anadenanthera peregrina*	Fabaceae	5	12.6	7–18
*Mimosa caesalpiniifolia*	Fabaceae	5	15.6	10–24
*Anacardium occidentale*	Anacardiaceae	4	14.3	13–15

^a^ Reported richness values are after subtracting compounds also found in blanks.

We used the Hapsite Headspace Sampling System (Inficon) in conjunction with the Hapsite to analyze food samples. The headspace attachment enables analysis of solid and liquid samples. Once collected, food samples were allowed to warm in the headspace at 80°C for 15 min prior to analysis. The headspace flow pressure was 80 kPa. Column specifications, run time, and temperature ramp were identical to the procedure for airborne samples.

### 2.4 Data analysis

For both applications, we utilized automatic peak detection *via* the SmartIQ software, followed by manual inspection of peaks (Drea et al., 2013). In cases where peaks overlapped or had poor resolution, the fragmented ions were extracted from the total ion chromatogram to identify individual components by both retention time and characteristic ion *m*/*z* values (Appendix S2). When possible, compound identity was tentatively determined through a National Institute of Standards (NIST) library search (Appendices S3 and S4), although many compounds from mammalian scent samples lack NIST matches (Charpentier, Barthes, Proffit, Bessière, & Grison, 2012; Drea et al., 2013; Kücklich et al., 2017; Weiß et al., 2018).

In addition to controls taken after every scent mark analysis, we also collected blanks of ambient air. We gathered *N* = 7 airprobe blanks, taken on average every 17.38 (±21.7*SD*) runs (including scent mark and control runs). Additionally, the airprobe purges sampled air from the line after each collection. For the headspace, blanks (*N* = 4) were collected on average every 6.4 (±9.9*SD*) headspace runs. The Hapsite also possesses a concentrator cleanout function that purges the device, which was performed nightly. Compounds present in blanks were removed from analyses (airprobe: *N* = 9 compounds; headspace: *N* = 40; Table 2; Appendices S3 and S4) (Charpentier et al., 2012; Drea et al., 2013).

**Table 2:** List of tentatively identified compounds from scent marks collected with the Hapsite airprobe, and the additional sample types they were found in. Full compound details are provided in Appendix S3

**Scent marks only**	
2,3‐butanedione, 2‐butanone, 3‐methyl	Ethyl acetate
4‐cyanocyclohexene	Furan, 2‐ethyl
Acetic acid, methyl ester	Methyltris(trimethylsiloxy)silane
Benzaldehyde	p‐cymene
Benzene bromopentafluro	Styrene
Ethanol	Terpene 1^a^
**Scent marks and controls**	
1,4‐pentadiene	Terpene 2^a^
Acetic acid	Terpene 3^a^
Anisole	Terpene 4^a^
Cyclohexane	Terpene 5^a^
Cyclotrisiloxane, hexamethyl	Terpene 6^a^
Ethylbenzene	Terpene 7^a^
Heptanal	Terpene 8^a^
Hexanal	Terpene 9^a^
n‐hexane	Xylene
Nonanal	
**Scent marks, controls and blanks**	
Benzene, 1,4‐dichloro	Tert‐butyldimethylsilanol
Benzoic acid, 2‐[(trimethylsilyl)oxy]‐ methyl ester	Toluene
Carbon dioxide	Trichloroethylene
Cyclotetrasiloxane, octamethyl	Trichloronitromethane
Heptane	

^a^ Numerous terpene compounds and isomers were detected in scent mark chromatograms. However, due to the similarity of their mass spectra fragmentation patterns, specific identification could not be determined, and therefore, they are identified numerically based on retention time.

#### 2.4.1 Application 1: Scent mark sampling

We conducted a one tailed paired *t* test to assess differences in chemical richness (number of compounds present) between scent marks and their matched controls. We expected that scent marks would have higher richness than controls, as both should contain baseline chemicals from the tree, but only scent marks would have added chemicals from animal secretions. The richness of scent secretions has proven to be an effective measure to differentiate biologically relevant behavioral and physiological variables in primates (e.g., Crawford & Drea, 2015; delBarco‐Trillo & Drea, 2014). We also conducted a discriminant function analysis (DFA) to determine whether scent marks displayed distinctive chemical signatures from controls. Summary statistics on compound consistency are reported in the results. For subsequent analyses, we excluded scent mark compounds that were present in matched controls.

To test the relationship between the chemical composition of scent marks and marmoset behavior, we conducted a two‐tailed Spearman's correlation between scent mark richness and (a) the number of scent marks placed on the same gouge hole within 48 hr, (b) total number of visits to the gouge hole (with and without scent marking) within 48 hr, (c) number of scent marks 48 hr prior to the sampled mark, (d) number of visits to the gouge hole (with and without scent marking) 48 hr prior to the sampled mark, and (e) gouge hole volume. Nonparametric statistics (i.e., Spearman's correlation) were employed because behavioral variables were not normally distributed. We did not find an effect of specific tree or gouge hole (with hole nested within tree ID) on scent mark richness (nested ANOVA: *F* = 0.93, *p* = 0.553), and so each scent mark was treated as an independent data point. As a follow‐up analysis, we also correlated gouge hole volume with the number of scent marks 48 hr prior to sampling, to test whether previous scent marks could influence the relationship between richness and gouge hole size. The time frame of 48 hr reflects best estimates of exudate flow and visitation rates to enable adequate sampling of revisits and remarks (Garber & Porter, 2010).

To reduce the large number of detected chemicals into representative variables that characterize scent profile variation, we conducted a principal component analysis (PCA) (Drea et al., 2013). Each chemical was recorded as a binary presence/absence variable within each sample. Since controlling sample volume in wild‐deposited scent marks will be unfeasible, this served as a more practical approach than attempting to estimate compounds’ relative abundance (but see discussion). Compounds found in *N* = 1 sample were excluded from the PCA (e.g., Spence‐Aizenberg et al., 2018; Zidat et al., 2018), resulting in *N* = 44 compounds included in the analysis. To determine the number of PCs to extract, we examined the scree plot, visualized a fit line through the majority of PCs with >1 eigenvalues and extracted PCs above this line (Jolliffe, 2002). This yielded one PC which explained 17.0% of the variance in scent mark chemical composition. We then conducted correlations between the extracted PC scores for each sampled gouge hole and (a) the total number of visits to the gouge hole 48 hr prior, (b) number of scent marks placed 48 hr prior, (c) total number of visits 48 hr after marking, (d) number of remarks 48 hr after marking, and (e) gouge hole volume. The extracted component contained *N* = 3 PC scores that were >3 standard deviations above the mean; we utilized Spearman's correlations (two‐tailed) to control for the effect of these large values.

#### 2.4.2 Application 2: Food odor sampling

To assess differences in chemical richness between exudate species, we conducted a one‐way ANOVA. To test for differences in the cumulative chemical profile of food samples, we followed the statistical approach of Nevo et al. (2016). We first conducted a PCA to generate representative variables characterizing scent profile variation, following the same procedure for scent marks. In total, *N* = 46 exudate compounds were entered into the PCA. Following the criteria above, we extracted *N* = 3 PCs. Cumulatively, these three PCs accounted for 37.1% of the total variance in exudate chemical composition. Winter, Dodou, and Wieringa (2009) found that such multivariate analyses are warranted in conditions with a very large number of input variables, high factor loadings, and extraction of a limited number of factors. The extraction of three PCs follows recommendations by Winter et al. (2009) specific for our sample size, variable number, and factor loadings. Finally, following Nevo et al. (2016), we conducted MANOVAs and DFA on these PCs to characterize differences in chemical signatures by species. As exudate analyses were based on somewhat low sample size, interpretations should be treated with caution. Nonetheless, these data can serve the aim of this study, to demonstrate the utility of portable GC‐MS for obtaining biologically relevant results on olfactory compounds.

## 3 RESULTS

### 3.1 Application 1: Scent mark sampling

#### 3.1.1 Chemical characteristics of scent marks

Scent marks had significantly higher richness than matched controls (*t*_63_ = 8.4, *p* < 0.001), indicating that scent mark samples detected compounds beyond the background environment. Subtracting matched control compounds eliminated $\bar{x}$ = 7.2 ± 2.3(*SD*) compounds from scent marks, with 43.8% of all detected compounds found in scent marks and controls. This high overlap indicates that controls captured many of the compounds from background environment also present in scent marks. These overlap compounds included known contaminants which were detected and eliminated from previous studies on mammalian olfaction (Table 2; Appendix S3). Lastly, DFA found a significant difference between the chemical signature of scent marks and controls (Wilk's *λ* = 0.3, *p* = 0.002), with 92.2% of samples correctly classified, indicating that scent marks displayed unique chemical profiles relative to controls.

Summary details on scent mark chemical characteristics are provided in Table 3. Of the compounds present in scent marks, a large proportion (40.5%) were found in only one scent mark. However, of the remaining compounds, there was relatively high consistency between samples (Table 3). Of the identified compounds, *N* = 8 matched substances previously reported as present in marmoset glandular secretions collected in captivity, demonstrating that the Hapsite was capable of detecting relevant compounds under field conditions (Appendix S3).

**Table 3:** Summary compound characteristics and consistency across samples for scent marks and exudates

Variable	Scent marks	Exudates
Total compounds in all samples	74	129
*N* (%) compounds in one sample	30 (40.5%)	83 (64.3%)
*N* (%) compounds in >10% of samples^a^	29 (65.9%)	46 (100%)^b^
*N* (%) compounds in >20% of samples^a^	17 (38.6%)	15 (32.6%)
*N* (%) identified compounds previously documented in similar sample types	8 (25.8%): common marmoset secretions^c^	6 (60.0%): plant spp.^c^

^a^ Of compounds found in >1 sample.

^b^ Of *N* = 19 exudate samples, >10% by default represents all compounds found in *N* > 1 sample.

^c^ Details on identified compounds are in Appendices S3 and S4; tallies exclude compounds found in blanks.

#### 3.1.2 Biological relevance to behavior

**FIGURE 2:**
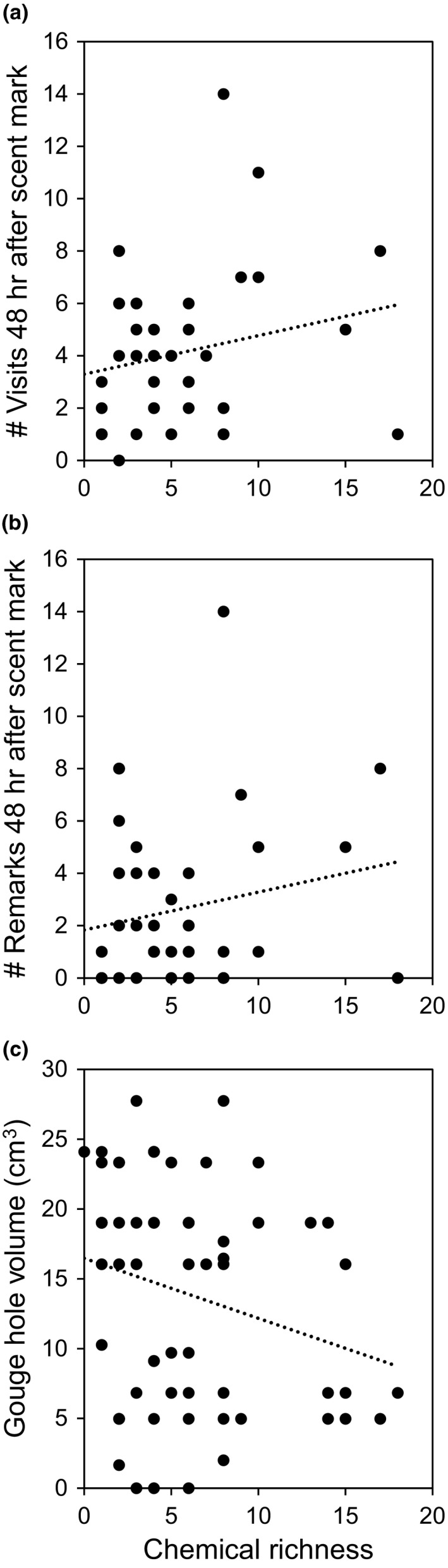
Relationship between chemical richness of scent marks and (a) the number of feeding visits to a gouge hole 48 hr after scent marking, (b) number of remarks placed on gouge hole within 48 hr of initial marking, and (c) gouge hole volume, an indicator of feeding use intensity. Dotted line is linear trendline

The chemical characteristics of scent marks displayed a relationship with variables indicative of feeding behavior (Table 4). There was a significant positive relationship between scent mark chemical richness and the number of total revisits and remarks by animals within 48 hr (Figure 2a,b; Table 4), with animals more often revisiting and remarking exudate holes that received richer scent cues. There was also a significant association between scent mark richness and the number of marks 48 hr prior, but not the total number of visits (Table 4). There was a significant negative relationship between gouge hole size and scent mark richness, largely driven by scent marks with the highest richness being deposited on smaller (i.e., newer) holes (Figure 2c). There was no association between gouge hole volume and the number of marks placed 48 hr prior (Table 4), suggesting this trend may reflect the composition of individual marks, rather than chemical accumulation from previous marks.

**Table 4:** Spearman's correlations between behavioral feeding variables and measures of scents’ chemical composition

Behavioral variable	Scent richness			Scent variation (PC1)		
	*ρ*	*p*	*df*	*ρ*	*p*	*df*
Visits after	**0.38**	**0.012**	42	0.21	0.163	42
Scent marks after	**0.48**	**0.001**	39	**0.38**	**0.014**	39
Visits before	−0.09	0.596	35	0.20	0.232	35
Scent marks before	**0.34**	**0.044**	33	**0.48**	**0.004**	33
Gouge hole volume	**−0.29**	**0.024**	58	**−0.41**	**0.001**	58

Note

Significant correlations (*p* < 0.05) shown in bold.

All before/after measures are within 48 hr of deposition of sampled scent mark. Richness is the number of compounds present after subtracting compounds found in matched controls and blanks.

The single extracted PC represents variation in the chemical profile of scent marks, by creating a single variable whose values reflect the spectrum of differing possible chemical components in a scent mark. This variation in chemical composition demonstrated a relationship with variables indicative of feeding behavior (Table 4). There was a significant relationship between the PC and gouge hole volume, and the number of scent marks placed both 48 hr prior and after sample deposition (Table 4). As positive or negative PC values reflect differing combinations of chemicals, these correlations can be interpreted as an association between marks with certain chemical compositions and feeding behavior. The PC did not show a relationship with total visits to the gouge hole (with and without scent marks) either 48 hr before marking or after (Table 4).

### 3.2 Application 2: Food odor sampling

#### 3.2.1 Exudates

Summary details on exudates’ chemical characteristics are provided in Table 3. Like scent marks, a large number of the present compounds only appeared in one sample (64.3%). Yet, the remaining compounds demonstrated some consistency across samples despite the high number of unique compounds. Several of the identified compounds are known to naturally occur in various plant species (Table 3; Appendix S4). The chemical richness of exudates did not differ between tree species (one‐way ANOVA: *F*_3,15_ = 0.33, *p* = 0.806), with species accounting for only 6.1% of the variation in chemical richness (Table 1).

**FIGURE 3:**
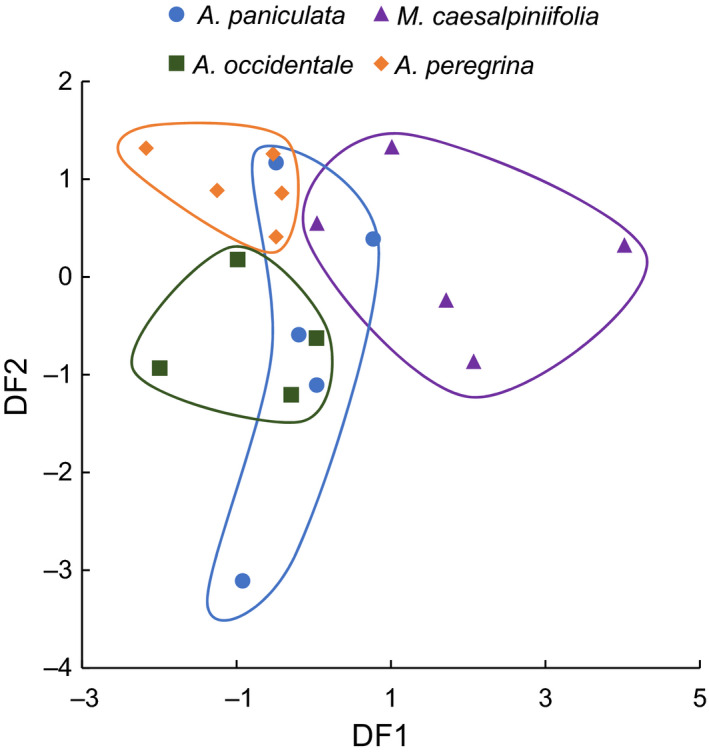
Discriminant functions of exudates plotted by species. Circles isolate the domain of species markers of the same color

Despite having similar richness values, the exudates of most species displayed distinctive chemical signatures (Figure 3). MANOVA detected significant differences in PC scores between species (*F*_9,32_ = 2.86, Wilk's *λ* = 0.20, *p* = 0.006), with species explaining 41.6% of PC score variation. Likewise, DFA showed distinct, but not always mutually exclusive, PC score domains by species (Figure 3) with 68.4% of samples being classified correctly. In particular, *Mimosa caesalpiniifolia*, *Anadenanthera peregrina*, and *Anacardium occidentale* had either exclusive or minimally overlapping chemical profiles, while *Acacia paniculata's* chemical profile displayed large overlap with other exudate species (Figure 3). Likewise, all three discriminant functions demonstrated significance, or trends toward significance (DF1: Wilk's *λ* = 0.20, *p* = 0.005; DF2: Wilk's *λ* = 0.51, *p* = 0.043; DF3: Wilk's *λ* = 0.79, *p* = 0.066).

## 4 DISCUSSION

### 4.1 Feasibility of portable GC‐MS to study chemical ecology in the field

Our aim was to test whether portable GC‐MS could be used under field conditions to gather biologically relevant data on mammalian olfaction. While we did encounter logistical challenges (discussed below), we were able to successfully conduct two example applications relevant to chemical ecology. The portable GC‐MS was able to differentiate olfactory signatures of exudate species, akin to previous studies investigating the chemical ecology of foraging (Nevo et al., 2016; Rodríguez et al., 2013). We were also able to characterize the chemical composition of scent marks deposited by wild, arboreal marmoset monkeys in their natural environment, which included compounds previously documented in marmoset glandular secretions. Moreover, we were able to relate the chemical composition of scent marks to measures of marmoset feeding behavior (visitation and marking of food sources, and food use intensity), demonstrating the biological relevance of these data. This represents an important methodological advancement, allowing us to link olfactory cues used by animals to their real‐time behavioral and ecological context.

Collecting scent samples from wild animals in the field poses unique challenges compared to research on captive or wild‐captured and anesthetized animals. Previous work validated the Hapsite portable GC‐MS as capable of detecting volatile organic compounds (but not higher boiling point compounds) present in captive marmoset odors that were analyzed via a standard benchtop GC‐MS device (Kücklich et al., 2017). Our study builds on this by using control samples of the background environment to isolate scent mark compounds placed by animals under wild conditions. Delineating the origin of compounds in mammalian chemical ecology studies is difficult (Charpentier et al., 2012), particularly in natural scenarios where plant‐based compounds may originate from either the natural substrate (i.e., tree), or as metabolized compounds from animals feeding on that tree. Nonetheless, our results indicate that meaningful scent signatures were isolated using this technique. This is suggested by the overall greater number of compounds present in scent marks relative to controls. Likewise, several of our detected substances were known components of marmoset glandular secretions. Also, the significant relationship between scent variation and aspects of subsequent feeding behavior suggest that the chemical signatures detected have biological relevance for marmosets. While there were a large number of compounds unique to only a single sample, the moderate consistency between the remaining compounds also points to samples coming from a consistent biological source (i.e., a marmoset). Previous work on owl monkey (*Aotus* spp.) glandular secretions found greater variability in scent samples from wild than captive animals, attributed to the effects of a broad ranging, individualized diet in the wild (Spence‐Aizenberg et al., 2018). Marmoset scent profiles are also known to vary with age, sex, and individual identity (Smith, 2006; Smith et al., 2001), and these factors likely contribute to the high variability (and number of unique compounds) in our data set, as well as in other studies of mammalian chemical ecology (Nair et al., 2018; Weiß et al., 2018).

### 4.2 Considerations for applying portable GC‐MS in the field

While our applications proved feasible, we did encounter some important limitations of portable GC‐MS that should be carefully considered prior to use. The degree to which these limitations may hinder data collection will vary extensively with field site, study species, and a project's research aims. The most critical consideration for researchers will likely be battery life, as this reduces the device's portability. The manufacturer reports battery life as 2–3 hr. While we did not fully expend our batteries during data collection, we lost 3%–4% of total battery life within 10–15 min, leading to a conservative estimate of 4.2 total hrs. Furthermore, the battery cannot be conserved by turning the device off between analyses (although it does have a lower power standby mode), since re‐heating the device significantly drains the battery. Carrying multiple, charged batteries may be helpful, but with the tradeoff of more bulk and weight (~3 kg per battery) to transport. It should also be noted that if the battery fails during analysis, data will be lost. Given these limitations, we recommend either (a) keeping the device at a central location with a power source and transporting it only to collect samples (our approach), or (b) keeping the device in standby mode while in the field, closely monitoring battery levels prior to collecting samples, and returning the device to a power source during analysis.

The feasibility of carrying the Hapsite for extended periods will likely depend on characteristics of the field site. For our site, with a dense forest understory and uneven terrain, carrying the device while simultaneously tracking and observing highly mobile animals proved difficult, even with a multi‐person field team. Likewise, field sites and study species that require additional equipment to obtain samples (e.g., ladders and/or ropes) may find that continuously carrying the device is not viable. However, the feasibility of this approach would improve considerably in more open habitats or where researchers can easily move equipment around a field site. Research on species that occupy small home ranges located near a power source would be ideal for using portable GC‐MS. While the addition of the headspace (necessary to analyze solids) is an impairment to the unit's portability, the device can still be readily transported to field sites and avoids the problems associated with sample degradation during storage and transport to the laboratory. For certain projects, in‐field analysis may also obviate the need for import and/or export permits.

Habitat type will also influence feasibility of accessing scents placed by wild animals. Sites and study species where sampling occurs on the ground or within standing height should not pose obstacles to the use of portable GC‐MS. However, when accessing greater heights, the feasibility of sampling will vary with forest structure. Nearby branches or tree trunks must be able to support the weight of the device and/or researcher, and be close enough to enable sampling. Indeed, we had the most difficulty in areas with only thin branches or lianas, irrespective of a sample's height in the canopy. Ideally, study animals should be sufficiently habituated for researchers to observe the exact location of scent marks. As such, researchers should consider their study conditions carefully to determine feasibility and recognize that all‐occurrence sampling may not be possible. Overall, our ability to obtain a relatively large number of samples from a highly mobile, arboreal primate in a tropical forest (across a range of canopy heights) is encouraging and suggests that accessing scent samples can be feasible even under challenging field conditions.

The abundance of compounds in scents has been shown to encode biologically relevant information (e.g., Smith, 2006). However, in this study, scent compounds were measured as either present or absent, due to the inability to control wild‐deposited sample volumes as well as the poorer sensitivity and detection limits of the Hapsite relative to benchtop GC‐MS instruments. Quantitation of scent peaks was attempted; however, the poor precision of the measurements made quantitative correlations difficult. Many of the peaks in samples, while above the detection limit, were below the acceptable quantitation limit of 10 times the background noise. Secondly, controlling sample volume was not reliable due to the variability of the gas‐phase sampling system of the instrument. Use of an internal standard compound would improve sampling precision; however, introducing such a standard would be challenging for most in‐field studies. However, it may be possible to quantify the relative abundance of compounds by comparing peak areas within a sample. This would allow the relative abundance of scent compounds to be compared between different scent samples, albeit from differing deposited volumes. In addition, the headspace sampling unit, which uses a sealed chamber to heat samples and collect a larger more concentrated sample, showed improved detection limits and may enhance the quantitative analysis. This sampling could be used when sufficient volumes of solid or liquid scent sources (i.e., feces and urine) can be obtained; alternately, scents could be analyzed along with their substrates (e.g., bark with a scent mark), while using controls to isolate compounds of interest.

### 4.3 Insights into marmoset olfaction

While testing the in‐field utility of portable GC‐MS to measure mammalian olfactory compounds was the primary goal of this study, preliminary insight into the chemical ecology of marmoset foraging can also be gained. Marmosets’ scent marking of gouged exudate holes demonstrates a connection with feeding behavior, although the exact nature of this relationship is not entirely straightforward. Scent marks with richer chemical cues had more subsequent feeding visits and remarks, suggesting a relationship between scent cues and the behavior of recipients. However, we found the opposite pattern between richness and gouge hole volume, with the richest scent marks being placed on smaller (i.e., newer), rather than larger (i.e., older) holes. Smaller holes were not associated with increased marking prior to sampling, suggesting that richer signals being placed on smaller holes were not a by‐product of previous marks. Likewise, the extracted PC showed a relationship with both gouge hole volume and the number of subsequent remarks, demonstrating that (a) the exact chemical components of marks varies between holes of different sizes and (b) marks with differing composition garner different levels of remarking behavior. Although contradictory, this could indicate an interplay between scent marking, the age of gouge holes, and frequency of use in which scent plays a role establishing new gouge hole feeding sites. Under this scenario, animals might place specific (including richer) scent cues on younger gouges. This parallels anecdotal field observations of frequent visitation and scent marking of newly created gouge holes (Thompson, pers. obs.). Additionally, marmosets may rely more heavily on visual, rather than olfactory, cues as gouge holes become larger. It is also possible that marmosets may be using secretions to ameliorate the challenges associated with gouging bark at new holes, however, the fact that the inner layers of bark are often more mechanically challenging than the outer layers (Thompson et al., 2014) suggests that this may not be the case. Despite the preliminary nature of these conclusions, the demonstrated ability to associate scent characteristics with the behavior of scent recipients represents an important step forward for understanding how animals use olfaction in ecologically relevant settings.

Our findings regarding the scent profiles of marmoset foods mirror previous work on plant volatile organic compounds (e.g., Nevo et al., 2016; Rodríguez et al., 2013). Exudates showed at least partially distinct chemical profiles. While we evaluated a limited number of species, these differences indicate the potential for marmosets to use scent when selecting foods, most likely in conjunction with visual cues (Melin et al., 2019; Nevo & Heymann, 2015).

## 5 CONCLUSIONS

While portable GC‐MS has limits, our data show that it presents a viable option to gather biologically meaningful data under field conditions. This represents an alternative to current laboratory‐based sampling methods which entail problematic storage and transportation issues (Drea et al., 2013; Nair et al., 2018; Spence‐Aizenberg et al., 2018). Likewise, it facilitates real‐time sampling that can link the chemical cues being sensed by wild animals with behavioral and ecological context, which is a novel application relative to sampling directly from the glands of restrained or anesthetized animals. While real‐time sampling via portable GC‐MS alleviates these issues, it also comes with its own set of trade‐offs. The Hapsite has a lesser ability to measure more stable compounds and can be less sensitive than benchtop models, which may lead to fewer total compounds being detected (Kücklich et al., 2017). Nonetheless, our data demonstrate that the device is sufficiently sensitive to detect compounds of interest in mammalian olfaction that display biological relevance to foraging behavior. The optimal methodology for field researchers will likely depend on a broad range of factors including the ability to properly store and transport samples, project‐specific needs to detect nonvolatile compounds, and conditions at the field site. However, our ability to conduct two example applications under relatively challenging field conditions bodes well for the versatility of using portable GC‐MS in the field.

## CONFLICTS OF INTEREST

The authors have no conflicts of interest to declare.

## AUTHOR CONTRIBUTION

AWL, CJV, and CLT conceived the ideas and designed methodology. CJV, CLT, KNB, LCOM, MABO collected data. AWL, CLT, and KNB analyzed data. CLT led writing of the manuscript. All authors contributed critically to drafts and gave final approval for publication.

## Supporting information

Supplementary Materials

## Data Availability

Data have been archived in Dryad (https://doi.org/10.5061/dryad.t4b8gthzc).
