# Supplementary material for: What smells? Developing in‐field methods to characterize the chemical composition of wild mammalian scent cues
Source: Ecol Evol. 2020 Apr 12;10(11):4691–701. doi: 10.1002/ece3.6224 (PMC7297786; doi:10.1002/ece3.6224)
Supplement: Supplementary file 1 — Supplementary Materials [file ECE3-10-4691-s001.pdf]

Appendix S1: Detailed Methods for Accessing Scent Marks in the Forest Canopy

To hoist the Hapsite into the forest canopy to collect samples, we utilized a two-rope system (Figure A1 A-D). The 'device rope' was fastened directly to the Hapsite, while the 'climbing rope' was used to hoist into the tree (Figure A1 A-B). For the device rope, we adapted field guidelines for firefighters (IAFC, 2004), with the final set up as follows: we first tied a figure eight on a bight in the middle of the rope (see Ashley, 1944 for knot descriptions). This was placed in the middle of the device's handle. Both ends of the rope from this knot were wrapped around the handle. At the end of the handle, we attached carabiners to an access point on the Hapsite, with the rope ends threaded through each carabineers and a figure eight stopper knot tied on the ends. The two ends were then tied together with a square knot. The excess ends of the rope were cut, taped, and melted to prevent fraying. The device rope remained fastened at all times to facilitate quick sampling, and did not interfere with the function of the Hapsite.

When hoisting the device, a climbing-grade carabineer connected the device rope to the climbing rope via a bight on each rope. The climbing rope (Figure A1 B) was placed in the tree either by throwing the line over the branch (with or without a separate throw line) from the ground or by placing the line over the branch from a ladder. We used a cambium saver over the top of the branch to reduce friction. The end of the climbing rope attached to the device rope was configured with a figure eight on a bight, with the climbing-grade carabiner attached to the bight and a figure eight stopper knot toward the near end of the rope. The cambium saver was placed on the opposite end of the figure eight. As with the device-rope, this configuration was fastened at all times to facilitate quick sampling. When placing the cambium saver over the branch, a slip knot was tied on the opposite end of the cambium saver (localizing the cambium saver between the figure eight and slip knot). Once over the branch, the slip knot easily pulls out

to free the climbing line. With the device rope attached to the climbing rope (through the bights in both ropes) via carabiner (Figure A1 C-D), the device was ready to be hoisted up by pulling the climbing line.

APPENDIX FIGURE A1: Configuration to hoist the Hapsite portable GC-MS into the forest canopy to sample scent marks. A. Detail of device rope configuration. B. Detail of climbing rope configuration with cambium saver. C. Connected device and climbing ropes, ready to hoist. D. Device hoisted in tree with researcher accessing sample via ladder.

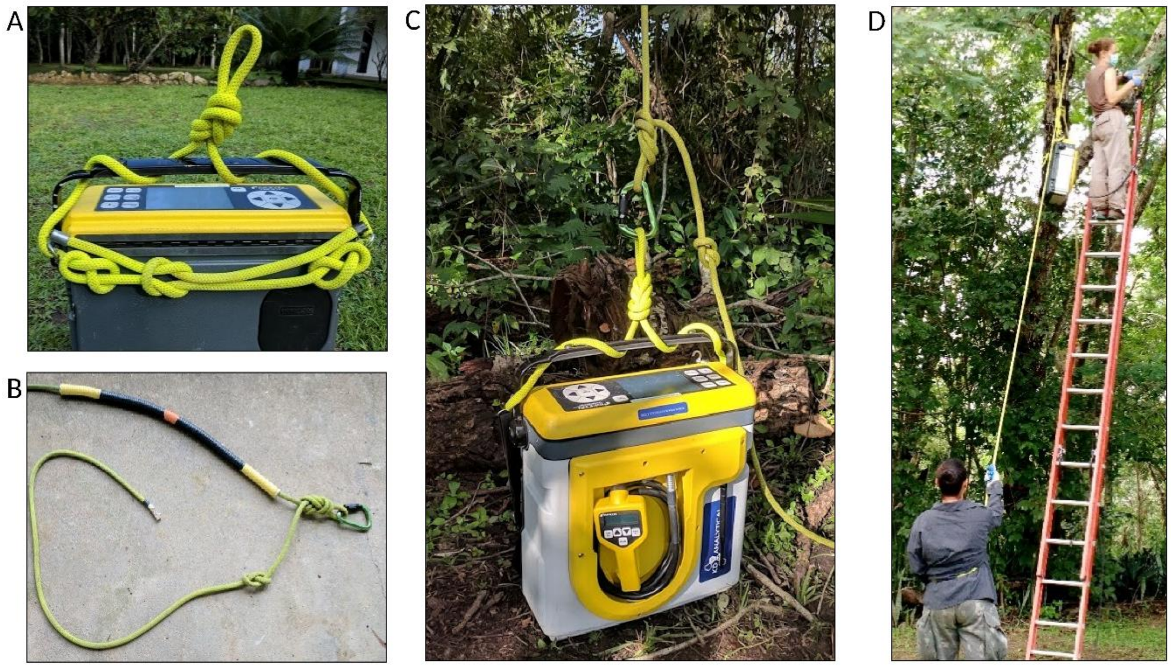

APPENDIX S2 A. Example chromatogram from a marmoset scent mark (black) with the paired control (blue) using the Hapsite airprobe. Initial truncated peak with large total ion count (TIC) identifies as carbon dioxide. B. Example chromatogram from an exudate sample analyzed with the headspace.

A.

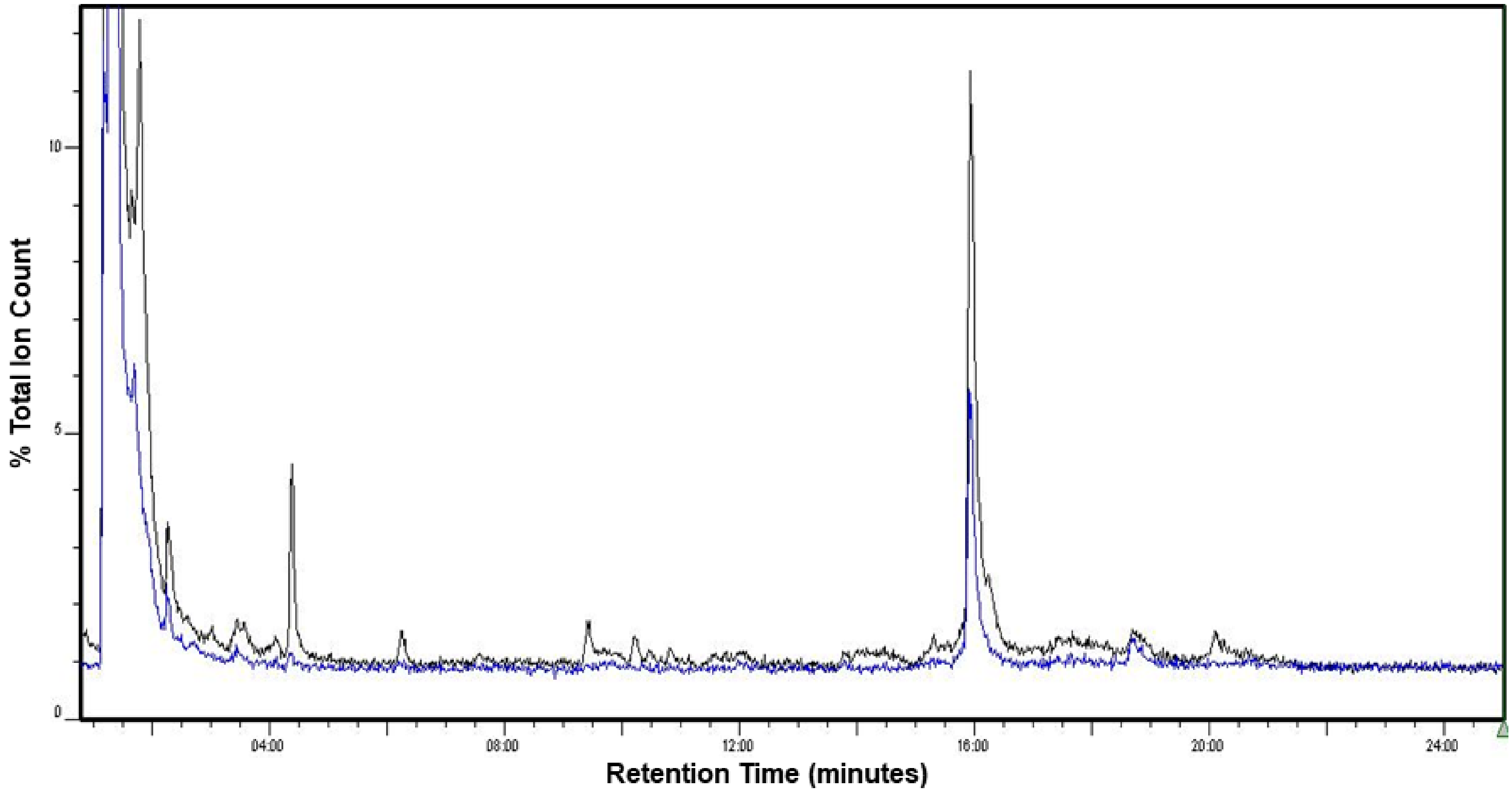

B.

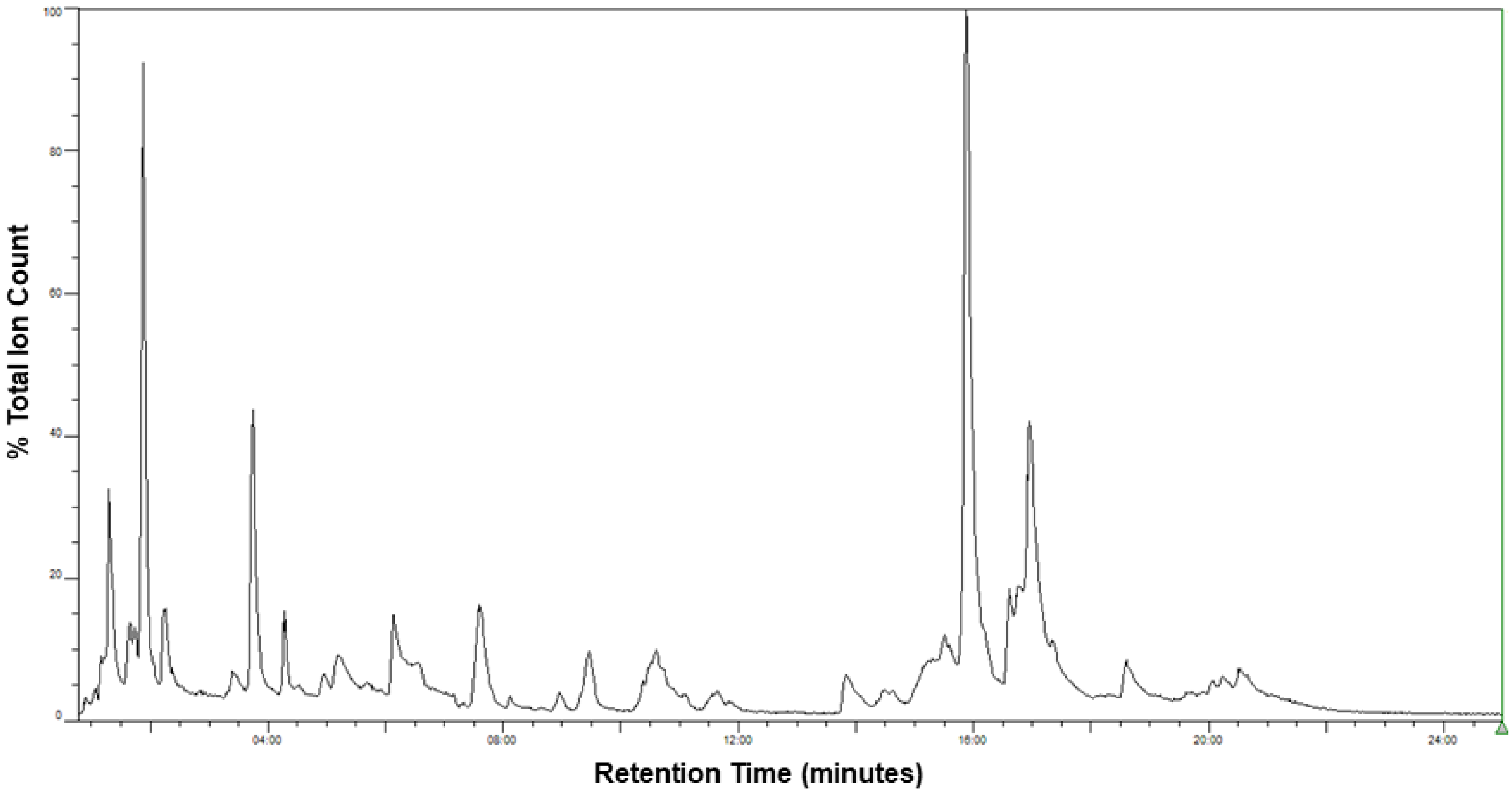
100
80
60
40
20
0
% Total Ion Count
04:00
08:00
12:00
16:00
20:00
24:00
Retention Time (minutes)

Appendix S3. List of tentatively identified compounds found in scent marks collected via the Hapsite airprobe. Compounds are displayed with the mean match factor (SI) across samples (out of 1000), retention time, compound class, and the sample set(s) it was found (SM=scent mark; C=control; B=blank). Pertinent references to previous scent studies or other documented origins are provided. *=Found in only one sample. †=Previously documented as present in marmoset secretions collected by directly sampling scent glands.

**Compound Identification**	**SI**	**Retention Time (min.)**	**Classification**	**Sample**	**Reference**
carbon dioxide	907	1.18	acidic oxide	SM, C, B	Burger 2005; Charpentier et al. 2012; Costello et al. 2014; PubChem 2019
ethanol†	581	1.65	alcohol	SM	Burger 2005; Costello et al. 2014; Kücklich et al. 2017
1,4-pentadiene	598	1.87	alkene	SM, C	Costello et al. 2014
acetic acid, methyl ester	637	1.92	ester	SM*	Costello et al. 2014
tert-butyldimethylsilanol	586	2.28	silanol	SM, C, B	Graiver et al. 2003
2,3-butanedione, 2-butanone, 3-methyl†	654	2.29	ketone	SM	Costello et al. 2014; Kücklich et al. 2017
n-hexane†	615	2.58	alkane	SM, C	Burger 2005; Kücklich et al. 2017
ethyl acetate	699	2.67	ester	SM	Costello et al. 2014
acetic acid†	680	2.74	carboxylic acid	SM, C	Burger 2005; Charpentier et al. 2012; Costello et al. 2014; Kücklich et al. 2017; Smith et al. 2001
trichloronitromethane	564	3.42	alkyl halide	SM, C, B	PubChem 2019
cyclohexane	646	3.50	alkane	SM, C	Costello et al. 2014; USDA 2019
trichloroethylene	585	4.08	alkyl halide	SM, C, B	PubChem 2019
furan, 2-ethyl†	651	4.12	alkane	SM*	Kücklich et al. 2017
heptane	619	4.34	alkane	SM, C, B	Charpentier et al. 2012; Costello et al. 2014
toluene	636	6.22	alkene	SM, C, B	Charpentier et al. 2012; Kücklich et al. 2017

hexanal†	637	7.46	alkanal	SM, C	Burger 2005; Charpentier et al. 2012; Costello et al. 2014; Kücklich et al. 2017; USDA 2019
cyclotrisiloxane, hexamethyl	657	9.38	cyclosiloxane	SM, C	Graiver et al. 2003
benzene bromopentafluro	559	10.20	alkyl halide	SM	PubChem 2019
ethylbenzene	634	10.39	aromatic hydrocarbon	SM, C	Costello et al. 2014; Kücklich et al. 2017; Pohanish & Sittig 2008
xylene	626	10.78	aromatic	SM, C	Charpentier et al. 2012; Costello et al. 2014
styrene	797	11.59	aromatic, terpene	SM	Charpentier et al. 2012; USDA 2019
heptanal	610	11.96	aldehyde, aromatic	SM, C	USDA 2019; Yannai 2004
anisole	715	12.46	aromatic, ester	SM, C	USDA 2019
terpene 1[a]	604	13.44	terpene	SM	Charpentier et al. 2012; USDA 2019
terpene 2[a]	666	13.78	terpene	SM, C	Charpentier et al. 2012; USDA 2019
benzaldehyde†	728	13.91	aldehyde, aromatic	SM	Burger 2005; Costello et al. 2014; Kücklich et al. 2017; Smith et al. 2001; Spence-Aizenberg et al. 2018; USDA 2019
terpene 3[a]	718	15.21	terpene	SM, C	Charpentier et al. 2012; USDA 2019
terpene 4[a]	566	15.25	terpene	SM, C	Charpentier et al. 2012; USDA 2019
terpene 5[a]	880	15.48	terpene	SM, C	Charpentier et al. 2012; USDA 2019
4-cyanocyclohexene	716	15.68	nitrile, cyclohexane	SM*	Sigma Aldrich 2019
benzene, 1,4-dichloro	752	15.88	aromatic	SM, C, B	Pohanish & Sittig 2008
terpene 6 [a]	699	16.33	terpene	SM, C	Costello et al. 2014
cyclotetrasiloxane, octamethyl	716	16.38	cyclosiloxane	SM, C, B	Graiver et al. 2003
terpene 7 [a]	688	16.91	terpene	SM, C	Charpentier et al. 2012; USDA 2019

terpene 8 [a]	738	17.35	terpene	SM, C	Charpentier et al. 2012; USDA 2019
terpene 9 [a]	579	17.67	terpene	SM, C	Charpentier et al. 2012; USDA 2019
p-cymene	654	18.21	terpene	SM	Burger 2005; Charpentier et al. 2012; Costello et al. 2014
methyltris(trimethylsiloxy)silane	631	18.33	organosilicon	SM*	Graiver et al. 2003
nonanal†	616	18.65	alkanal	SM, C	Kücklich et al. 2017; Burger 2005, Costello et al. 2014; USDA, 2019
benzoic acid, 2-[(trimethylsilyl)oxy]- methyl ester	608	20.13	organosilicon	SM, C, B	Graiver et al. 2003

[a] Numerous terpene compounds and isomers were detected in the chromatograms for the scent marks. However, due to the similarity of their mass spectra fragmentation patterns, specific identification could not be determined, and therefore they are identified numerically based on retention time

Appendix S4. List of tentatively identified compounds found in exudate samples collected via the Hapsite headspace unit. Compounds are displayed with the mean match factor (SI) across samples (out of 1000), retention time, compound class, and the sample set(s) it was found (E=exudate; B=blank). Pertinent references for compound origins are provided. *=Found in only one sample. Compounds with reference USDA (2019) have been previously documented to occur naturally in plants.

**Compound Identification**	**SI**	**Retention Time (min.)**	**Classification**	**Sample**	**Reference**
acetaldehyde	792	1.45	aldehyde, carbonyl	E	USDA 2019
dimethyl ether	830	1.65	ether	E	PubChem 2019
acetone	817	1.71	ketone	E, B	PubChem 2019; USDA 2019
heptane	827	1.87	alkane	E*	Charpentier et al. 2012; USDA 2019
carbon disulfide	795	2.01	organosulfur	E, B	Sigma-Aldrich 2019
1-propanol	806	2.12	alcohol	E*	USDA 2019
silanol, trimethyl	824	2.23	organosilicon	E, B	Graiver et al. 2003
2,3-butanedione	783	2.27	carbonyl	E	USDA 2019; Yannai 2004
benzene	774	3.02	aromatic hydrocarbon	E, B	Pohanish & Sittig 2008; USDA 2019
benzene, -fluoro	719	3.49	aromatic	E	PubChem 2019
trichloroethylene	747	4.03	organohalogen	E, B	PubChem 2019; Pohanish & Sittig 2008
hexane, 3-methyl	727	4.28	alkane	E, B	PubChem 2019
toluene	907	6.11	alkene	E, B	Charpentier et al. 2012; PubChem 2019; USDA 2019
2-pentene, 4,4-dimethyl	708	7.54	alkene	E	Costello et al. 2014; PubChem 2019
acetic acid	709	9.06	carboxylic acid	E	USDA 2019

cyclotrisiloxane, hexamethyl	782	9.31	organosilicon	E, B	Graiver et al. 2003
ethylbenzene	775	10.34	aromatic hydrocarbon	E, B	Pohanish & Sittig 2008
benzene, 1,3-dimethyl	848	10.68	aromatic hydrocarbon	E, B	Pohanish & Sittig 2008
heptanal	776	11.84	aldehyde, carbonyl	E	USDA 2019; Yannai 2004
benzaldehyde	790	13.83	aldehyde, aromatic	E, B	Pohanish & Sittig 2008; USDA 2019
benzaldehyde, oxime (z)	712	14.46	benzaldehyde	E, B	PubChem 2019
3-ethytoluene	728	15.49	aromatic hydrocarbon	E, B	PubChem 2019
benzene, 1,3-dichloro	851	15.86	aromatic	E, B	Pohanish & Sittig 2008
benzene, 1,4-dichloro	869	16.61	aromatic	E, B	Pohanish & Sittig 2008
benzene, 1,2-dichloro	788	16.73	aromatic	E, B	Pohanish & Sittig 2008
eucalyptol	838	16.94	cyclic ether, monoterpenoid	E	USDA 2019; Yannai 2004
3-carene	888	17.35	terpene	E, B	PubChem 2019; USDA 2019
nonanal	780	18.58	alkanal	E, B	Kücklich et al. 2017; Burger 2005, Costello et al. 2014; USDA 2019

References

Ashley, C. W. (1944). The Ashley book of knots. New York, NY: Doubleday.

Burger, B. V. (2005). Mammalian semiochemicals. In S. Schulz (Ed.), The chemistry of pheromones and other semiochemicals III (pp. 231–278). Berlin, Heidelberg: Springer, Berlin Heidelberg.

Charpentier, M. J. E., Barthes, N., Proffit, M., Bessière, J. M., & Grison, C. (2012). Critical thinking in the chemical ecology of mammalian communication: Roadmap for future studies. Functional Ecology, 26, 769–774.

Costello, B. L., Amann, A., Al-Kateb, H., Flynn, C., Filipiak, W., Khalid, T., … Ratcliffe, N. M. (2014). A review of the volatiles from the healthy human body. Journal of Breath Research, 8(1), 014001. https://doi.org/10.1088/1752-7155/8/1/014001

Graiver, D., Farminer, K., & Narayan, R. (2003). A review of the fate and effects of silicones in the environment. Journal of Polymers and the Environment, 11, 129.

International Association of Fire Chiefs (2004). Fundamentals of fire fighter skills. Sudbury, MA: Jones & Bartlett Learning.

Kücklich, M., Möller, M., Marcillo, A., Einspanier, A., Weiβ, B. M., Birkemeyer, C., & Widdig, A. (2017). Different methods for volatile sampling in mammals. PLoS ONE, 12, e0183440.

Pohanish, R. P., & Sittig, M. (2008). Sittig's handbook of toxic and hazardous chemicals and carcinogens, 5th ed. Norwich, NY: William Andrew Inc.

PubChem Database (2019). National Center for Biotechnology Information. Retrieved from https://pubchem.ncbi.nlm.nih.gov

Sigma-Aldrich (2019). GC analysis of volatiles in purple nitrile gloves on SPB-HAP after collection/desorption using Carbotrap 300 Tube. Darmstadt, Germany: Merck KGaA. Retrieved from https://www.sigmaaldrich.com/technical-documents/articles/analytical-applications/gc/gc-analysis-of-volatiles-in-purple-nitrile-gloves-g005933.html

Smith, T. E., Tomlinson, A. J., Mlotkiewicz, J. A., & Abbott, D. H. (2001). Female marmoset monkeys (Callithrix jacchus) can be identified from the chemical composition of their scent marks. Chemical Senses, 26, 449–458.

Spence-Aizenberg, A., Kimball, B. A., Williams, L. E., & Fernandez-Duque, E. (2018). Chemical composition of glandular secretions from a pair-living monogamous primate: Sex, age, and gland differences in captive and wild owl monkeys (Aotus spp.). American Journal of Primatology, 80, e22730

U.S. Department of Agriculture, Agricultural Research Service (2019). Dr. Duke's Phytochemical and Ethnobotanical Databases. Home Page, http://phytochem.nal.usda.gov/; https://doi.org/10.15482/USDA.ADC/1239279

Yannai, S. (2004). Dictionary of food compounds with CD-ROM: Additives, flavors, and ingredients. Boca Raton, FL: Chapman & Hall/CRC
